# Our New Department

**Published:** 1871-03

**Authors:** 


### PART III.

### Biographical, Ethical, Hygenic, and Miscellaneous.

### OUR NEW DEPARTMENT.

We again call attention to this new feature of our Journal. Our publisher has kindly consented to give sixteen additional number of pages for this purpose—making the number sixty-four, instead of forty-eight. We will appropriate this space to many things. We expect to give prominence to Biography, Ethics and Hygiene, but shall not confine ourselves to these exclusively. In other words, we intend to devote this department of the Journal to a multitude of subjects, of interest and value, more or less closely allied to the profession: subjects which we hope will prove beneficial to the practitioner—especially the young—to aid him, in meeting the wants of the people among whom he may labor. Believing this feature of the COMPANION a good one, we hope our professional brethren will appreciate and bestow that attention to it, which we feel assured will result in good to all—both to the old and young.

---

### OUR CORRESPONDENT—DR. W. A. N.

We call attention to some extracts from a letter from an old and talented member of the profession. Professional men, in these "latter days," more than ever, are environed by difficulties. It is a sad commentary upon the professional demoralization and degeneracy of the times, when the old men—ripe in age—feel it necessary to take up the pen in vindication of those principles by which professional gentlemen should be controlled. There is great wrong somewhere, and when the old men have passed away, who among the young of to-day will battle for the right? Who among them shall wear the mantle of honor dropping from the shoulders of the moral heroes of the past? Shall the flood of demoralization sweep everything before it?

The troubles of the true professional man are daily increasing. They are rife in every section, and prevalent in every community. It takes a moral hero to oppose and overcome them. Many, rather than contend for the right, either join hand in hand with the enemies of truth, or seal their lips, as the lesser evil, in order to avoid the animosity of those who damn all whom they cannot bend to their purposes. Principle is put in the balance with gain —and gain, however short-lived, throws the beam. They yield to conscious wrong for the present, at the sacrifice of the future. Demoralization is not wholly the offspring of the vicious and the vile—the weakness and supineness of the good, easy, negative character, gives energy and force to its infernal sweep to ruin. Where none will oppose, all must be engulphed.

Our correspondent alludes to several things of the weightiest interest to the profession. Singly and alone, no one man, however bold or able, can meet them. The profession, as a unit, must come up to the help of the truth, against the mighty. Like him, we think the Georgia Medical Association has a great and glorious work to accomplish. We do not hesitate to give our opinion that the Association, at its next meeting, should take steps to carry into practical operation the report of its Committee, which was adopted at Savannah, "on the relations of charlatans and their nostrums to legitimate medicine." The necessity of some action, in this direction, is felt on every side; and if, as suggested in that report, "an appropriate, brief address to the people, to be disseminated in the form of a tract," could be placed in the hands of every man and woman in the State, much good would result in dissipating the errors so prevalent in the minds of the people.

The Association should not stop here. But it should settle forever the question of charlantry among so-called professional men—men with diplomas, who either practice quackery, or countenance irregulars—thus sacrificing the honor and dignity of the profession, in pandering to selfishness or in circumventing their nobler brethren, by bidding for practice.

### OUR CORRESPONDENT, DR. T. O. C.

We are glad to know that our friend, Dr. T. O. C. sanctions our course in not publishing his previous article, for the reasons given. We are, however, equally glad to have the opportunity of publishing an article from his pen, as it contains valuable thoughts and suggestions for the well-being of the profession, and does not contain even one personal allusion. We are happy to state for the benefit of our friend and others, that the entire profession, as manifested in the medical societies of every section, are being aroused to the importance and necessity of establishing some uniform system, in regard to medical education and medical colleges, upon which all, both the profession and colleges, may unite. At the last annual session of the American Medical Association, a resolution was adopted declaring that it had the "power to control the subject of medical education in the United States;" and "the power to exercise that control in any manner upon which it may become agreed."

The Association having declared this power, it will become necessary for that body to agree and announce the requirements of the Colleges that are recognized by it, or recognized by the State Associations that are represented in it. We are glad to inform our friend that the Georgia Medical Association is already committed upon this subject. We, however, know, from its past history, what its action would be, without this committal. At the last meeting of the Association, the following resolution was adopted:

"*Resolved*, That we, of the Georgia Medical Association, will co-operate with our State Societies, and the American Medical Association, in raising the standard of preliminary and medical education."

In reply to the doubt which seemed to be cast upon the Assosiation, by our correspondent, as to the willingness of the Association to sustain the right, we think its past history affords ample response. In its action in regard to the case of Dr. Ramsey, many years ago, the principle of protecting the character of its private members, when professionally assailed, was established, which was reiterated by resolution at Savannah, 1869, in the following words:

"*Resolved*, That as this Association was instituted for the purpose of cultivating the science of Medicine and the collateral branches, as well as the promoting of good friendship among the members, that hereafter the Ga. Medical Association will not entertain or take cognizance of any differences or disputes between local or county medical societies, or between the Medical Colleges of the State, only so far as to decide matters of etiquette or violations of the Code of Ethics between its individual members."

The only construction to be properly placed upon the above language, is this: 1st. That where local societies, medical colleges or individual members of the profession, have individual and personal quarrels, involving no principle in the ethics; where all parties have equal standing in the profession, that the Association will not allow such parties to thrust themselves or their quarrels upon the time and consideration of the Association.

2. But when the differences of medical gentlemen, societies or colleges become of such a character as to involve the principles of the code of ethics, then the Association stands pledged to decide who is right or wrong. We do not hesitate to affirm that the past history of the Association gives ample security to the profession of the State—that no individual, however assailed or "surrounded by enemies," need apprehend any fear that the Association will not sustain him in defending and upholding the principles upon which it is governed—viz : the code of ethics. In the language of one of its old and distinguished members, uttered a few years ago, we, too, assert our belief that "the Georgia Medical Association will never, no, never, stultify itself" by endorsing wrong over right. Composed of gentlemen alive to honor and the true interests of the profession it cannot suffer its untarnished reputation to be prostituted to the endorsement of error, or the overthrow of principle. It will not ignore the demands of justice and right—predicated upon its own action—at the instigation of "wolves in sheep's clothing," or lower its proud ensign at the bidding of "ringery and kinery."

